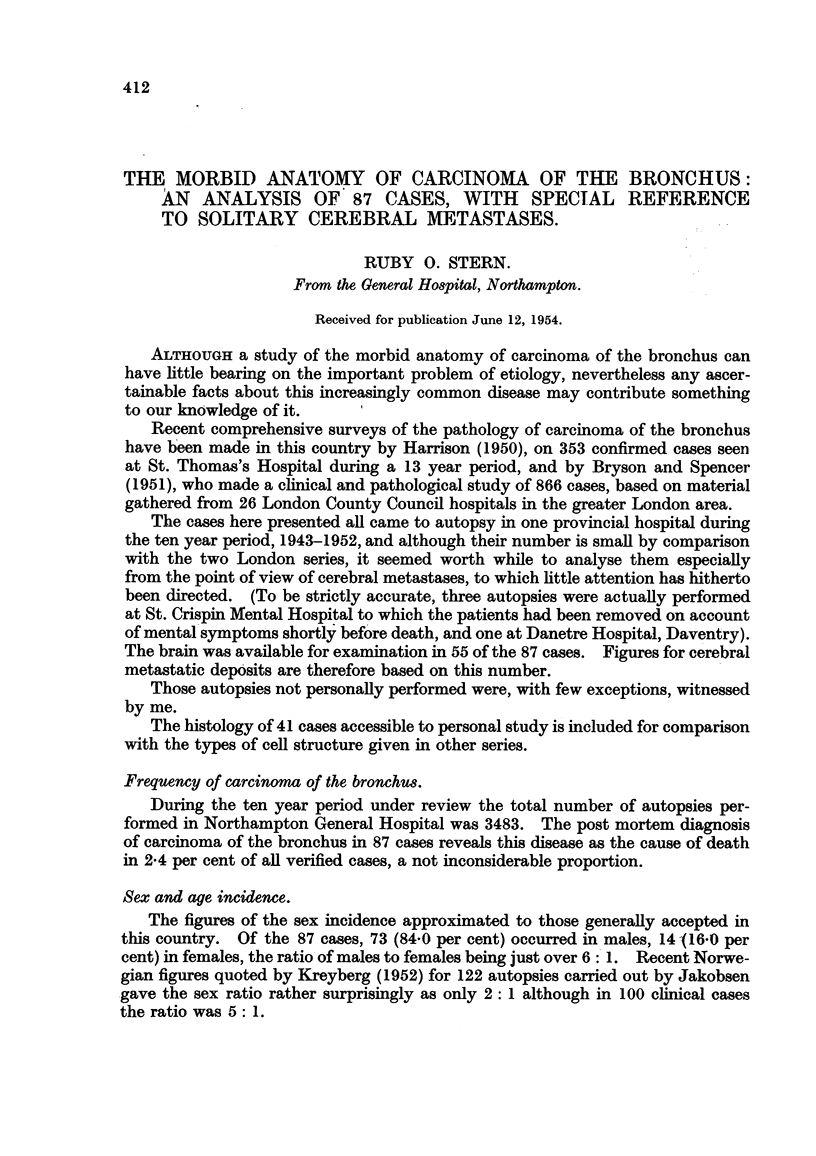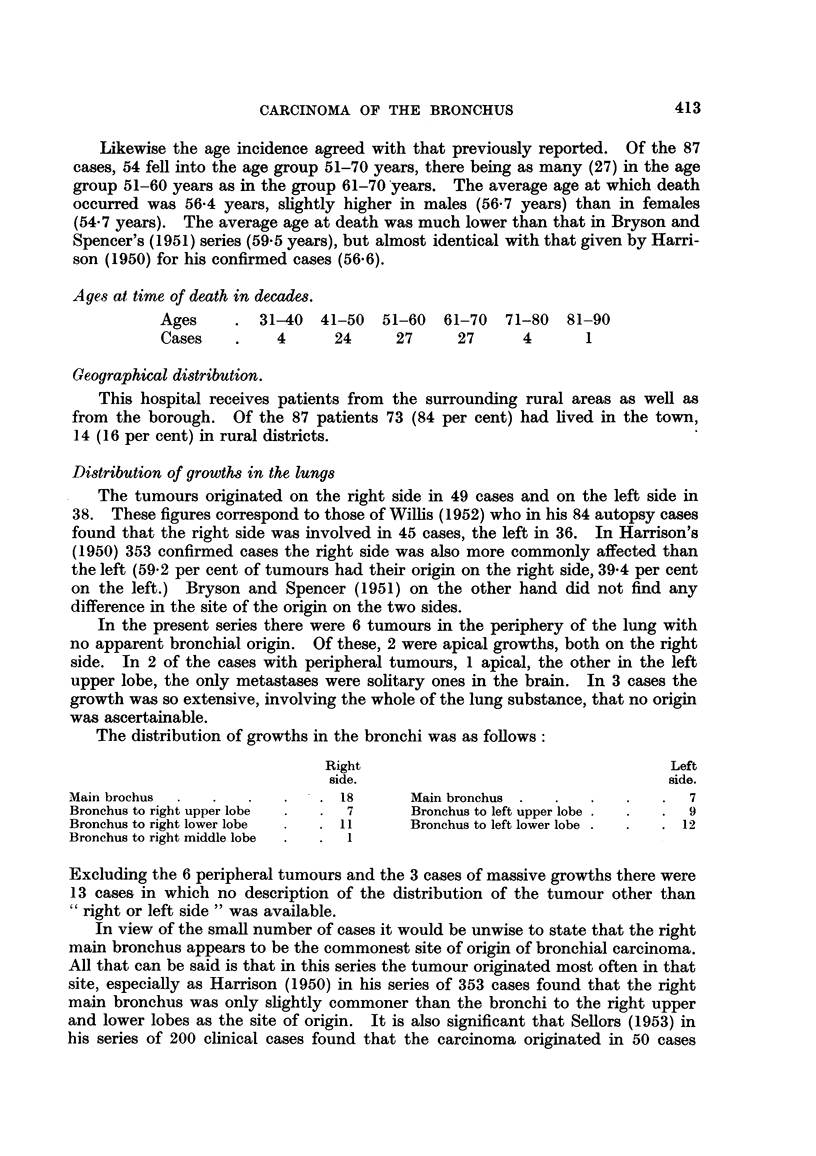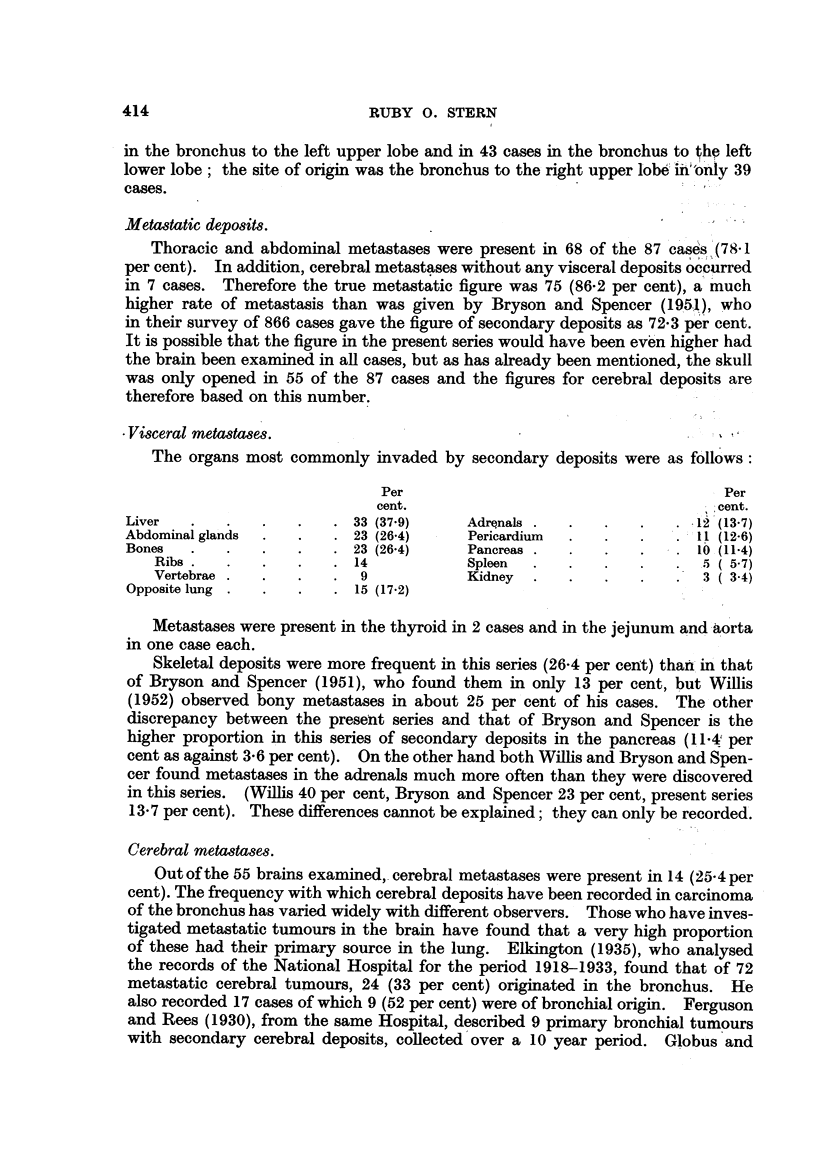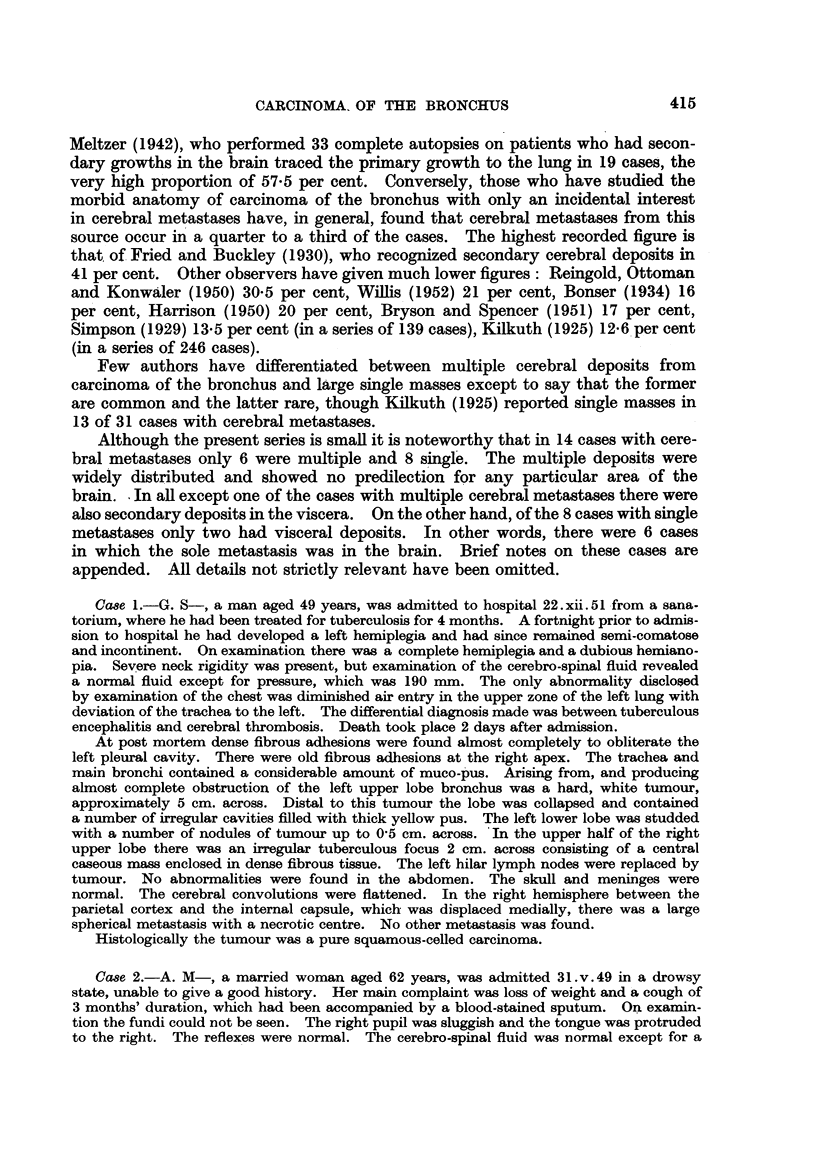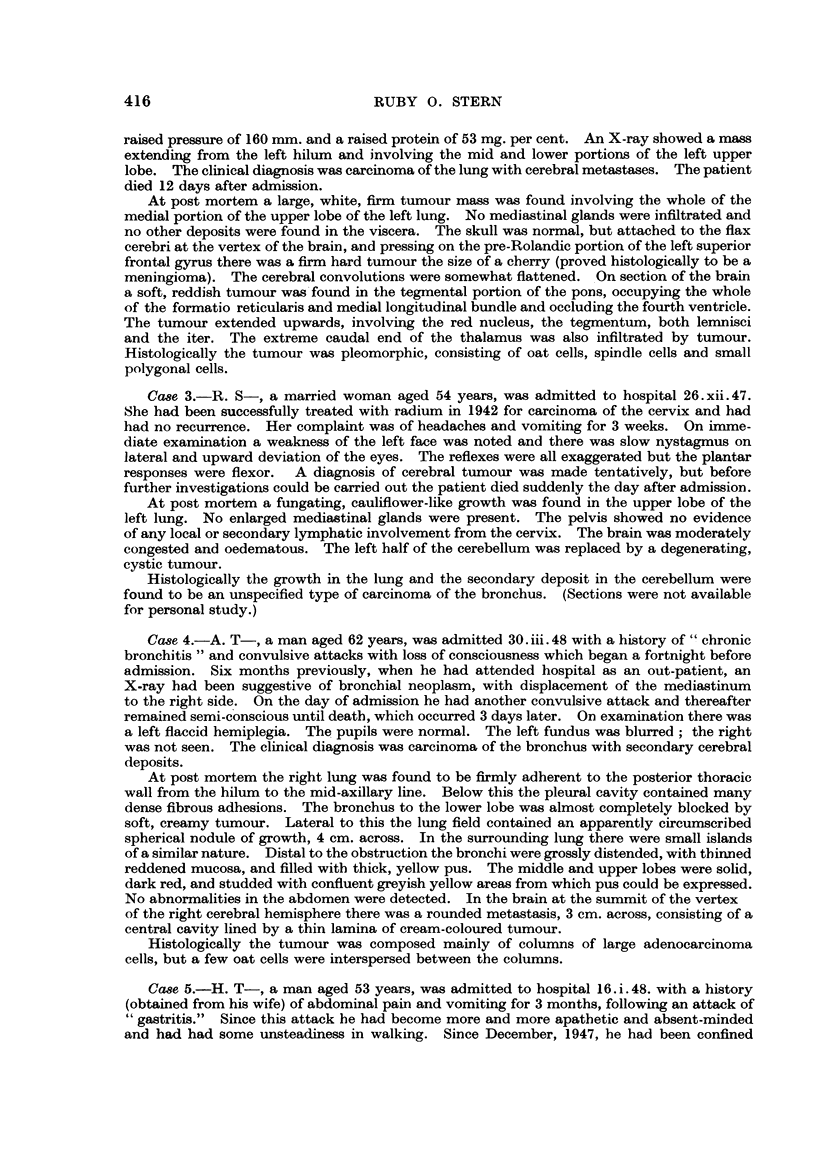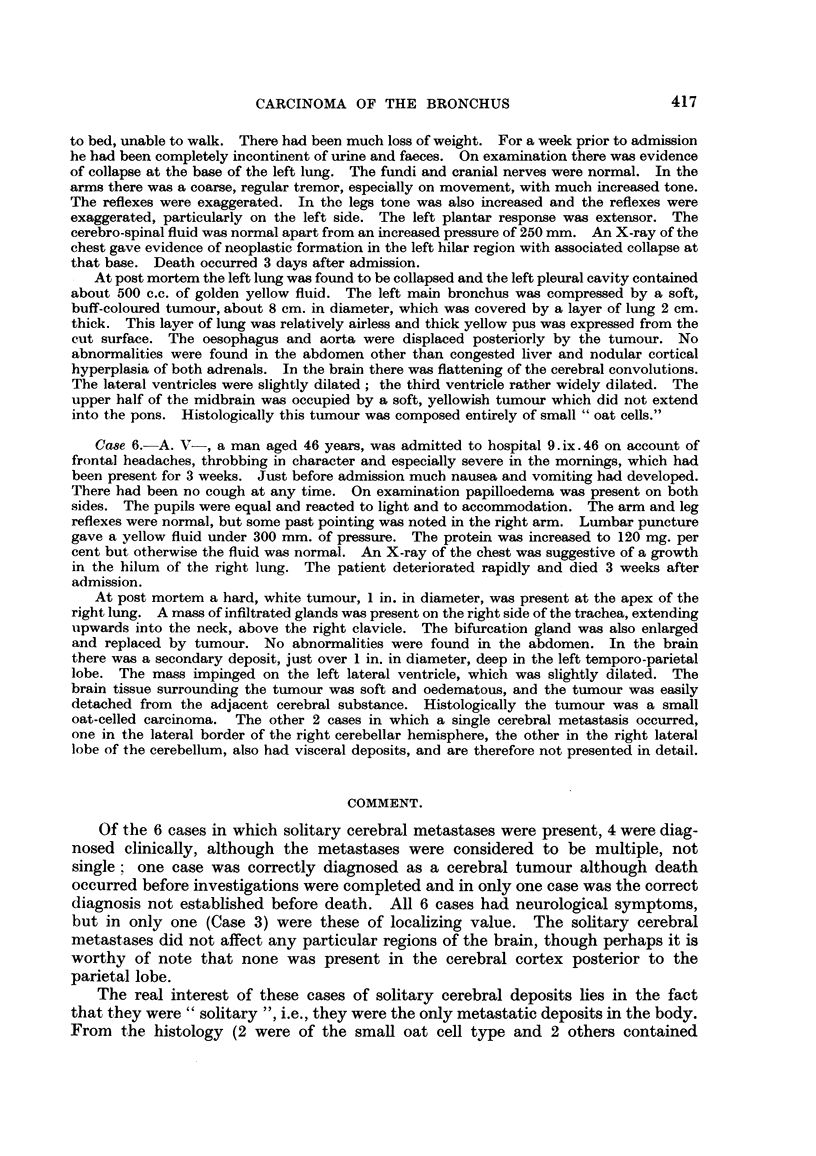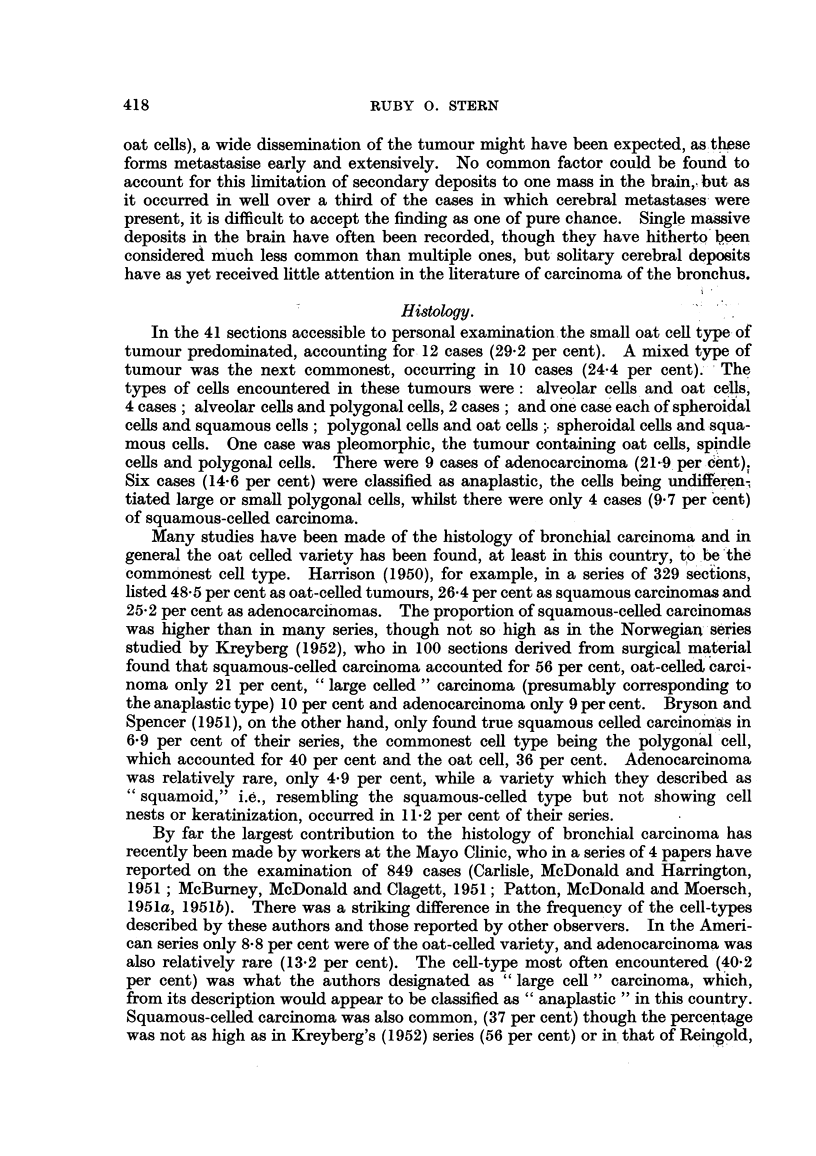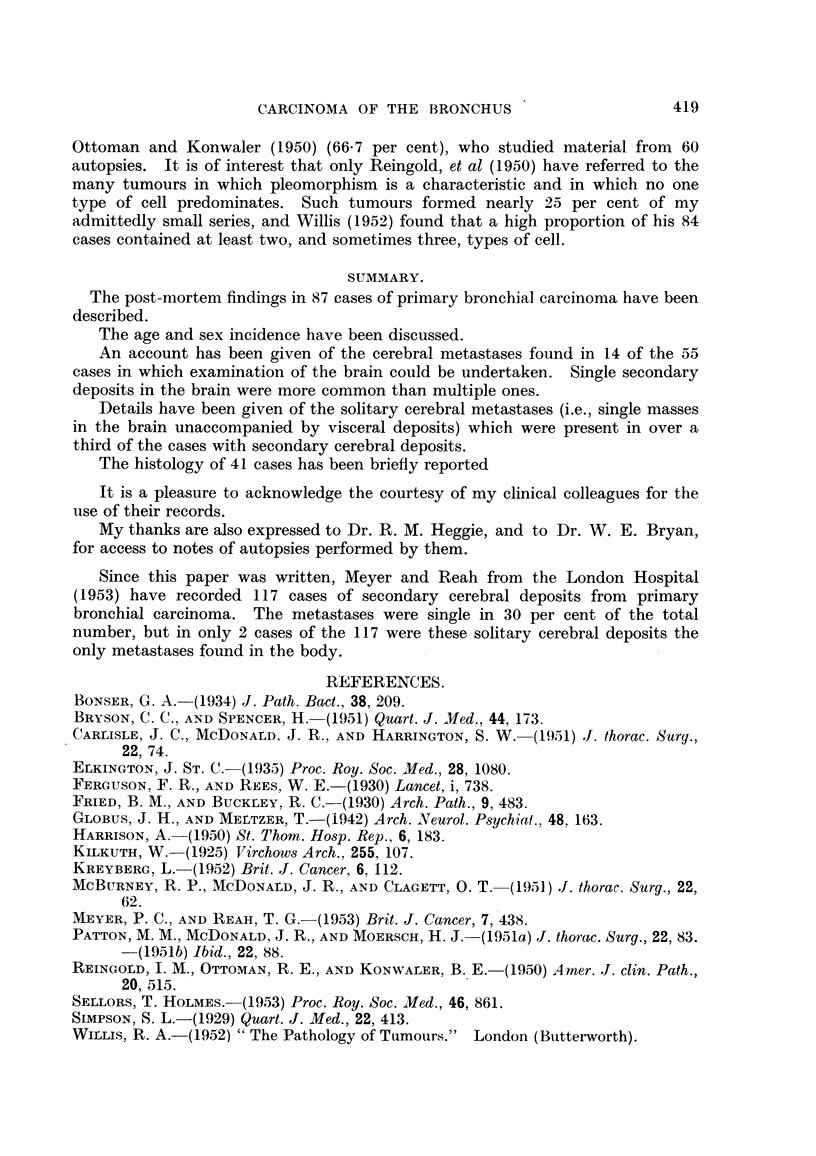# The Morbid Anatomy of Carcinoma of the Bronchus: An Analysis of 87 Cases, with Special Reference to Solitary Cerebral Metastases

**DOI:** 10.1038/bjc.1954.42

**Published:** 1954-09

**Authors:** Ruby O. Stern


### THE MORBID ANATOMY OF CARCINOMA OF THE BRONCHUS: AN ANALYSIS OF 87 CASES, WITH SPECIAL REFERENCE TO SOLITARY CEREBRAL METASTASES.

RUBY O. STERN.

*From the General Hospital, Northampton.*

Received for publication June 12, 1954.

ALTHOUGH a study of the morbid anatomy of carcinoma of the bronchus can have little bearing on the important problem of etiology, nevertheless any ascertainable facts about this increasingly common disease may contribute something to our knowledge of it.

Recent comprehensive surveys of the pathology of carcinoma of the bronchus have been made in this country by Harrison (1950), on 353 confirmed cases seen at St. Thomas's Hospital during a 13 year period, and by Bryson and Spencer (1951), who made a clinical and pathological study of 866 cases, based on material gathered from 26 London County Council hospitals in the greater London area.

The cases here presented all came to autopsy in one provincial hospital during the ten year period, 1943–1952, and although their number is small by comparison with the two London series, it seemed worth while to analyse them especially from the point of view of cerebral metastases, to which little attention has hitherto been directed. (To be strictly accurate, three autopsies were actually performed at St. Crispin Mental Hospital to which the patients had been removed on account of mental symptoms shortly before death, and one at Danetre Hospital, Daventry). The brain was available for examination in 55 of the 87 cases. Figures for cerebral metastatic deposits are therefore based on this number.

Those autopsies not personally performed were, with few exceptions, witnessed by me.

The histology of 41 cases accessible to personal study is included for comparison with the types of cell structure given in other series.

*Frequency of carcinoma of the bronchus.*

During the ten year period under review the total number of autopsies performed in Northampton General Hospital was 3483. The post mortem diagnosis of carcinoma of the bronchus in 87 cases reveals this disease as the cause of death in 2·4 per cent of all verified cases, a not inconsiderable proportion.

*Sex and age incidence.*

The figures of the sex incidence approximated to those generally accepted in this country. Of the 87 cases, 73 (84·0 per cent) occurred in males, 14 (16·0 per cent) in females, the ratio of males to females being just over 6 : 1. Recent Norwegian figures quoted by Kreyberg (1952) for 122 autopsies carried out by Jakobsen gave the sex ratio rather surprisingly as only 2 : 1 although in 100 clinical cases the ratio was 5 : 1.

Likewise the age incidence agreed with that previously reported. Of the 87 cases, 54 fell into the age group 51–70 years, there being as many (27) in the age group 51–60 years as in the group 61–70 years. The average age at which death occurred was 56·4 years, slightly higher in males (56·7 years) than in females (54·7 years). The average age at death was much lower than that in Bryson and Spencer's (1951) series (59·5 years), but almost identical with that given by Harrison (1950) for his confirmed cases (56·6).

*Ages at time of death in decades.*

| Ages | 31–40 | 41–50 | 51–60 | 61–70 | 71–80 | 81–90 |
|---|---|---|---|---|---|---|
| Cases | 4 | 24 | 27 | 27 | 4 | 1 |

*Geographical distribution.*

This hospital receives patients from the surrounding rural areas as well as from the borough. Of the 87 patients 73 (84 per cent) had lived in the town, 14 (16 per cent) in rural districts.

*Distribution of growths in the lungs*

The tumours originated on the right side in 49 cases and on the left side in 38. These figures correspond to those of Willis (1952) who in his 84 autopsy cases found that the right side was involved in 45 cases, the left in 36. In Harrison's (1950) 353 confirmed cases the right side was also more commonly affected than the left (59·2 per cent of tumours had their origin on the right side, 39·4 per cent on the left.) Bryson and Spencer (1951) on the other hand did not find any difference in the site of the origin on the two sides.

In the present series there were 6 tumours in the periphery of the lung with no apparent bronchial origin. Of these, 2 were apical growths, both on the right side. In 2 of the cases with peripheral tumours, 1 apical, the other in the left upper lobe, the only metastases were solitary ones in the brain. In 3 cases the growth was so extensive, involving the whole of the lung substance, that no origin was ascertainable.

The distribution of growths in the bronchi was as follows :

| | Right side. | | Left side. |
|---|---|---|---|
| Main brochus | 18 | Main bronchus | 7 |
| Bronchus to right upper lobe | 7 | Bronchus to left upper lobe | 9 |
| Bronchus to right lower lobe | 11 | Bronchus to left lower lobe | 12 |
| Bronchus to right middle lobe | 1 | | |

Excluding the 6 peripheral tumours and the 3 cases of massive growths there were 13 cases in which no description of the distribution of the tumour other than " right or left side " was available.

In view of the small number of cases it would be unwise to state that the right main bronchus appears to be the commonest site of origin of bronchial carcinoma. All that can be said is that in this series the tumour originated most often in that site, especially as Harrison (1950) in his series of 353 cases found that the right main bronchus was only slightly commoner than the bronchi to the right upper and lower lobes as the site of origin. It is also significant that Sellors (1953) in his series of 200 clinical cases found that the carcinoma originated in 50 cases in the bronchus to the left upper lobe and in 43 cases in the bronchus to the left lower lobe ; the site of origin was the bronchus to the right upper lobe in only 39 cases.

*Metastatic deposits.*

Thoracic and abdominal metastases were present in 68 of the 87 cases (78·1 per cent). In addition, cerebral metastases without any visceral deposits occurred in 7 cases. Therefore the true metastatic figure was 75 (86·2 per cent), a much higher rate of metastasis than was given by Bryson and Spencer (1951), who in their survey of 866 cases gave the figure of secondary deposits as 72·3 per cent. It is possible that the figure in the present series would have been even higher had the brain been examined in all cases, but as has already been mentioned, the skull was only opened in 55 of the 87 cases and the figures for cerebral deposits are therefore based on this number.

*Visceral metastases.*

The organs most commonly invaded by secondary deposits were as follows :

| | | Per cent. | | | Per cent. |
|---|---|---|---|---|---|
| Liver | 33 | (37·9) | Adrenals | 12 | (13·7) |
| Abdominal glands | 23 | (26·4) | Pericardium | 11 | (12·6) |
| Bones | 23 | (26·4) | Pancreas | 10 | (11·4) |
| Ribs | 14 | | Spleen | 5 | ( 5·7) |
| Vertebrae | 9 | | Kidney | 3 | ( 3·4) |
| Opposite lung | 15 | (17·2) | | | |

Metastases were present in the thyroid in 2 cases and in the jejunum and aorta in one case each.

Skeletal deposits were more frequent in this series (26·4 per cent) than in that of Bryson and Spencer (1951), who found them in only 13 per cent, but Willis (1952) observed bony metastases in about 25 per cent of his cases. The other discrepancy between the present series and that of Bryson and Spencer is the higher proportion in this series of secondary deposits in the pancreas (11·4 per cent as against 3·6 per cent). On the other hand both Willis and Bryson and Spencer found metastases in the adrenals much more often than they were discovered in this series. (Willis 40 per cent, Bryson and Spencer 23 per cent, present series 13·7 per cent). These differences cannot be explained ; they can only be recorded.

*Cerebral metastases.*

Out of the 55 brains examined, cerebral metastases were present in 14 (25·4 per cent). The frequency with which cerebral deposits have been recorded in carcinoma of the bronchus has varied widely with different observers. Those who have investigated metastatic tumours in the brain have found that a very high proportion of these had their primary source in the lung. Elkington (1935), who analysed the records of the National Hospital for the period 1918–1933, found that of 72 metastatic cerebral tumours, 24 (33 per cent) originated in the bronchus. He also recorded 17 cases of which 9 (52 per cent) were of bronchial origin. Ferguson and Rees (1930), from the same Hospital, described 9 primary bronchial tumours with secondary cerebral deposits, collected over a 10 year period. Globus and

Meltzer (1942), who performed 33 complete autopsies on patients who had secondary growths in the brain traced the primary growth to the lung in 19 cases, the very high proportion of 57·5 per cent. Conversely, those who have studied the morbid anatomy of carcinoma of the bronchus with only an incidental interest in cerebral metastases have, in general, found that cerebral metastases from this source occur in a quarter to a third of the cases. The highest recorded figure is that of Fried and Buckley (1930), who recognized secondary cerebral deposits in 41 per cent. Other observers have given much lower figures : Reingold, Ottoman and Konwaler (1950) 30·5 per cent, Willis (1952) 21 per cent, Bonser (1934) 16 per cent, Harrison (1950) 20 per cent, Bryson and Spencer (1951) 17 per cent, Simpson (1929) 13·5 per cent (in a series of 139 cases), Kilkuth (1925) 12·6 per cent (in a series of 246 cases).

Few authors have differentiated between multiple cerebral deposits from carcinoma of the bronchus and large single masses except to say that the former are common and the latter rare, though Kilkuth (1925) reported single masses in 13 of 31 cases with cerebral metastases.

Although the present series is small it is noteworthy that in 14 cases with cerebral metastases only 6 were multiple and 8 single. The multiple deposits were widely distributed and showed no predilection for any particular area of the brain. In all except one of the cases with multiple cerebral metastases there were also secondary deposits in the viscera. On the other hand, of the 8 cases with single metastases only two had visceral deposits. In other words, there were 6 cases in which the sole metastasis was in the brain. Brief notes on these cases are appended. All details not strictly relevant have been omitted.

*Case* 1.—G. S—, a man aged 49 years, was admitted to hospital 22.xii.51 from a sanatorium, where he had been treated for tuberculosis for 4 months. A fortnight prior to admission to hospital he had developed a left hemiplegia and had since remained semi-comatose and incontinent. On examination there was a complete hemiplegia and a dubious hemianopia. Severe neck rigidity was present, but examination of the cerebro-spinal fluid revealed a normal fluid except for pressure, which was 190 mm. The only abnormality disclosed by examination of the chest was diminished air entry in the upper zone of the left lung with deviation of the trachea to the left. The differential diagnosis made was between tuberculous encephalitis and cerebral thrombosis. Death took place 2 days after admission.

At post mortem dense fibrous adhesions were found almost completely to obliterate the left pleural cavity. There were old fibrous adhesions at the right apex. The trachea and main bronchi contained a considerable amount of muco-pus. Arising from, and producing almost complete obstruction of the left upper lobe bronchus was a hard, white tumour, approximately 5 cm. across. Distal to this tumour the lobe was collapsed and contained a number of irregular cavities filled with thick yellow pus. The left lower lobe was studded with a number of nodules of tumour up to 0·5 cm. across. In the upper half of the right upper lobe there was an irregular tuberculous focus 2 cm. across consisting of a central caseous mass enclosed in dense fibrous tissue. The left hilar lymph nodes were replaced by tumour. No abnormalities were found in the abdomen. The skull and meninges were normal. The cerebral convolutions were flattened. In the right hemisphere between the parietal cortex and the internal capsule, which was displaced medially, there was a large spherical metastasis with a necrotic centre. No other metastasis was found.

Histologically the tumour was a pure squamous-celled carcinoma.

*Case* 2.—A. M—, a married woman aged 62 years, was admitted 31.v.49 in a drowsy state, unable to give a good history. Her main complaint was loss of weight and a cough of 3 months' duration, which had been accompanied by a blood-stained sputum. On examintion the fundi could not be seen. The right pupil was sluggish and the tongue was protruded to the right. The reflexes were normal. The cerebro-spinal fluid was normal except for a raised pressure of 160 mm. and a raised protein of 53 mg. per cent. An X-ray showed a mass extending from the left hilum and involving the mid and lower portions of the left upper lobe. The clinical diagnosis was carcinoma of the lung with cerebral metastases. The patient died 12 days after admission.

At post mortem a large, white, firm tumour mass was found involving the whole of the medial portion of the upper lobe of the left lung. No mediastinal glands were infiltrated and no other deposits were found in the viscera. The skull was normal, but attached to the flax cerebri at the vertex of the brain, and pressing on the pre-Rolandic portion of the left superior frontal gyrus there was a firm hard tumour the size of a cherry (proved histologically to be a meningioma). The cerebral convolutions were somewhat flattened. On section of the brain a soft, reddish tumour was found in the tegmental portion of the pons, occupying the whole of the formatio reticularis and medial longitudinal bundle and occluding the fourth ventricle. The tumour extended upwards, involving the red nucleus, the tegmentum, both lemnisci and the iter. The extreme caudal end of the thalamus was also infiltrated by tumour. Histologically the tumour was pleomorphic, consisting of oat cells, spindle cells and small polygonal cells.

*Case* 3.—R. S—, a married woman aged 54 years, was admitted to hospital 26.xii.47. She had been successfully treated with radium in 1942 for carcinoma of the cervix and had had no recurrence. Her complaint was of headaches and vomiting for 3 weeks. On immediate examination a weakness of the left face was noted and there was slow nystagmus on lateral and upward deviation of the eyes. The reflexes were all exaggerated but the plantar responses were flexor. A diagnosis of cerebral tumour was made tentatively, but before further investigations could be carried out the patient died suddenly the day after admission.

At post mortem a fungating, cauliflower-like growth was found in the upper lobe of the left lung. No enlarged mediastinal glands were present. The pelvis showed no evidence of any local or secondary lymphatic involvement from the cervix. The brain was moderately congested and oedematous. The left half of the cerebellum was replaced by a degenerating, cystic tumour.

Histologically the growth in the lung and the secondary deposit in the cerebellum were found to be an unspecified type of carcinoma of the bronchus. (Sections were not available for personal study.)

*Case* 4.—A. T—, a man aged 62 years, was admitted 30.iii.48 with a history of " chronic bronchitis " and convulsive attacks with loss of consciousness which began a fortnight before admission. Six months previously, when he had attended hospital as an out-patient, an X-ray had been suggestive of bronchial neoplasm, with displacement of the mediastinum to the right side. On the day of admission he had another convulsive attack and thereafter remained semi-conscious until death, which occurred 3 days later. On examination there was a left flaccid hemiplegia. The pupils were normal. The left fundus was blurred ; the right was not seen. The clinical diagnosis was carcinoma of the bronchus with secondary cerebral deposits.

At post mortem the right lung was found to be firmly adherent to the posterior thoracic wall from the hilum to the mid-axillary line. Below this the pleural cavity contained many dense fibrous adhesions. The bronchus to the lower lobe was almost completely blocked by soft, creamy tumour. Lateral to this the lung field contained an apparently circumscribed spherical nodule of growth, 4 cm. across. In the surrounding lung there were small islands of a similar nature. Distal to the obstruction the bronchi were grossly distended, with thinned reddened mucosa, and filled with thick, yellow pus. The middle and upper lobes were solid, dark red, and studded with confluent greyish yellow areas from which pus could be expressed. No abnormalities in the abdomen were detected. In the brain at the summit of the vertex of the right cerebral hemisphere there was a rounded metastasis, 3 cm. across, consisting of a central cavity lined by a thin lamina of cream-coloured tumour.

Histologically the tumour was composed mainly of columns of large adenocarcinoma cells, but a few oat cells were interspersed between the columns.

*Case* 5.—H. T—, a man aged 53 years, was admitted to hospital 16.i.48. with a history (obtained from his wife) of abdominal pain and vomiting for 3 months, following an attack of " gastritis." Since this attack he had become more and more apathetic and absent-minded and had had some unsteadiness in walking. Since December, 1947, he had been confined to bed, unable to walk. There had been much loss of weight. For a week prior to admission he had been completely incontinent of urine and faeces. On examination there was evidence of collapse at the base of the left lung. The fundi and cranial nerves were normal. In the arms there was a coarse, regular tremor, especially on movement, with much increased tone. The reflexes were exaggerated. In the legs tone was also increased and the reflexes were exaggerated, particularly on the left side. The left plantar response was extensor. The cerebro-spinal fluid was normal apart from an increased pressure of 250 mm. An X-ray of the chest gave evidence of neoplastic formation in the left hilar region with associated collapse at that base. Death occurred 3 days after admission.

At post mortem the left lung was found to be collapsed and the left pleural cavity contained about 500 c.c. of golden yellow fluid. The left main bronchus was compressed by a soft, buff-coloured tumour, about 8 cm. in diameter, which was covered by a layer of lung 2 cm. thick. This layer of lung was relatively airless and thick yellow pus was expressed from the cut surface. The oesophagus and aorta were displaced posteriorly by the tumour. No abnormalities were found in the abdomen other than congested liver and nodular cortical hyperplasia of both adrenals. In the brain there was flattening of the cerebral convolutions. The lateral ventricles were slightly dilated ; the third ventricle rather widely dilated. The upper half of the midbrain was occupied by a soft, yellowish tumour which did not extend into the pons. Histologically this tumour was composed entirely of small " oat cells."

*Case* 6.—A. V—, a man aged 46 years, was admitted to hospital 9.ix.46 on account of frontal headaches, throbbing in character and especially severe in the mornings, which had been present for 3 weeks. Just before admission much nausea and vomiting had developed. There had been no cough at any time. On examination papilloedema was present on both sides. The pupils were equal and reacted to light and to accommodation. The arm and leg reflexes were normal, but some past pointing was noted in the right arm. Lumbar puncture gave a yellow fluid under 300 mm. of pressure. The protein was increased to 120 mg. per cent but otherwise the fluid was normal. An X-ray of the chest was suggestive of a growth in the hilum of the right lung. The patient deteriorated rapidly and died 3 weeks after admission.

At post mortem a hard, white tumour, 1 in. in diameter, was present at the apex of the right lung. A mass of infiltrated glands was present on the right side of the trachea, extending upwards into the neck, above the right clavicle. The bifurcation gland was also enlarged and replaced by tumour. No abnormalities were found in the abdomen. In the brain there was a secondary deposit, just over 1 in. in diameter, deep in the left temporo-parietal lobe. The mass impinged on the left lateral ventricle, which was slightly dilated. The brain tissue surrounding the tumour was soft and oedematous, and the tumour was easily detached from the adjacent cerebral substance. Histologically the tumour was a small oat-celled carcinoma. The other 2 cases in which a single cerebral metastasis occurred, one in the lateral border of the right cerebellar hemisphere, the other in the right lateral lobe of the cerebellum, also had visceral deposits, and are therefore not presented in detail.

### COMMENT.

Of the 6 cases in which solitary cerebral metastases were present, 4 were diagnosed clinically, although the metastases were considered to be multiple, not single ; one case was correctly diagnosed as a cerebral tumour although death occurred before investigations were completed and in only one case was the correct diagnosis not established before death. All 6 cases had neurological symptoms, but in only one (Case 3) were these of localizing value. The solitary cerebral metastases did not affect any particular regions of the brain, though perhaps it is worthy of note that none was present in the cerebral cortex posterior to the parietal lobe.

The real interest of these cases of solitary cerebral deposits lies in the fact that they were " solitary ", i.e., they were the only metastatic deposits in the body. From the histology (2 were of the small oat cell type and 2 others contained oat cells), a wide dissemination of the tumour might have been expected, as these forms metastasise early and extensively. No common factor could be found to account for this limitation of secondary deposits to one mass in the brain, but as it occurred in well over a third of the cases in which cerebral metastases were present, it is difficult to accept the finding as one of pure chance. Single massive deposits in the brain have often been recorded, though they have hitherto been considered much less common than multiple ones, but solitary cerebral deposits have as yet received little attention in the literature of carcinoma of the bronchus.

*Histology.*

In the 41 sections accessible to personal examination the small oat cell type of tumour predominated, accounting for 12 cases (29·2 per cent). A mixed type of tumour was the next commonest, occurring in 10 cases (24·4 per cent). The types of cells encountered in these tumours were: alveolar cells and oat cells, 4 cases; alveolar cells and polygonal cells, 2 cases; and one case each of spheroidal cells and squamous cells; polygonal cells and oat cells; spheroidal cells and squamous cells. One case was pleomorphic, the tumour containing oat cells, spindle cells and polygonal cells. There were 9 cases of adenocarcinoma (21·9 per cent). Six cases (14·6 per cent) were classified as anaplastic, the cells being undifferentiated large or small polygonal cells, whilst there were only 4 cases (9·7 per cent) of squamous-celled carcinoma.

Many studies have been made of the histology of bronchial carcinoma and in general the oat celled variety has been found, at least in this country, to be the commonest cell type. Harrison (1950), for example, in a series of 329 sections, listed 48·5 per cent as oat-celled tumours, 26·4 per cent as squamous carcinomas and 25·2 per cent as adenocarcinomas. The proportion of squamous-celled carcinomas was higher than in many series, though not so high as in the Norwegian series studied by Kreyberg (1952), who in 100 sections derived from surgical material found that squamous-celled carcinoma accounted for 56 per cent, oat-celled carcinoma only 21 per cent, " large celled " carcinoma (presumably corresponding to the anaplastic type) 10 per cent and adenocarcinoma only 9 per cent. Bryson and Spencer (1951), on the other hand, only found true squamous celled carcinomas in 6·9 per cent of their series, the commonest cell type being the polygonal cell, which accounted for 40 per cent and the oat cell, 36 per cent. Adenocarcinoma was relatively rare, only 4·9 per cent, while a variety which they described as " squamoid," i.e., resembling the squamous-celled type but not showing cell nests or keratinization, occurred in 11·2 per cent of their series.

By far the largest contribution to the histology of bronchial carcinoma has recently been made by workers at the Mayo Clinic, who in a series of 4 papers have reported on the examination of 849 cases (Carlisle, McDonald and Harrington, 1951; McBurney, McDonald and Clagett, 1951; Patton, McDonald and Moersch, 1951*a*, 1951*b*). There was a striking difference in the frequency of the cell-types described by these authors and those reported by other observers. In the American series only 8·8 per cent were of the oat-celled variety, and adenocarcinoma was also relatively rare (13·2 per cent). The cell-type most often encountered (40·2 per cent) was what the authors designated as " large cell " carcinoma, which, from its description would appear to be classified as " anaplastic " in this country. Squamous-celled carcinoma was also common, (37 per cent) though the percentage was not as high as in Kreyberg's (1952) series (56 per cent) or in that of Reingold,

Ottoman and Konwaler (1950) (66·7 per cent), who studied material from 60 autopsies. It is of interest that only Reingold, *et al* (1950) have referred to the many tumours in which pleomorphism is a characteristic and in which no one type of cell predominates. Such tumours formed nearly 25 per cent of my admittedly small series, and Willis (1952) found that a high proportion of his 84 cases contained at least two, and sometimes three, types of cell.

### SUMMARY.

The post-mortem findings in 87 cases of primary bronchial carcinoma have been described.

The age and sex incidence have been discussed.

An account has been given of the cerebral metastases found in 14 of the 55 cases in which examination of the brain could be undertaken. Single secondary deposits in the brain were more common than multiple ones.

Details have been given of the solitary cerebral metastases (i.e., single masses in the brain unaccompanied by visceral deposits) which were present in over a third of the cases with secondary cerebral deposits.

The histology of 41 cases has been briefly reported

It is a pleasure to acknowledge the courtesy of my clinical colleagues for the use of their records.

My thanks are also expressed to Dr. R. M. Heggie, and to Dr. W. E. Bryan, for access to notes of autopsies performed by them.

Since this paper was written, Meyer and Reah from the London Hospital (1953) have recorded 117 cases of secondary cerebral deposits from primary bronchial carcinoma. The metastases were single in 30 per cent of the total number, but in only 2 cases of the 117 were these solitary cerebral deposits the only metastases found in the body.

### REFERENCES.

Bonser, G. A.—(1934) *J. Path. Bact.*, **38**, 209.
Bryson, C. C., and Spencer, H.—(1951) *Quart. J. Med.*, **44**, 173.
Carlisle, J. C., McDonald, J. R., and Harrington, S. W.—(1951) *J. thorac. Surg.*, **22**, 74.
Elkington, J. St. C.—(1935) *Proc. Roy. Soc. Med.*, **28**, 1080.
Ferguson, F. R., and Rees, W. E.—(1930) *Lancet*, i, 738.
Fried, B. M., and Buckley, R. C.—(1930) *Arch. Path.*, **9**, 483.
Globus, J. H., and Meltzer, T.—(1942) *Arch. Neurol. Psychiat.*, **48**, 163.
Harrison, A.—(1950) *St. Thom. Hosp. Rep.*, **6**, 183.
Kilkuth, W.—(1925) *Virchows Arch.*, **255**, 107.
Kreyberg, L.—(1952) *Brit. J. Cancer*, **6**, 112.
McBurney, R. P., McDonald, J. R., and Clagett, O. T.—(1951) *J. thorac. Surg.*, **22**, 62.
Meyer, P. C., and Reah, T. G.—(1953) *Brit. J. Cancer*, **7**, 438.
Patton, M. M., McDonald, J. R., and Moersch, H. J.—(1951*a*) *J. thorac. Surg.*, **22**, 83.
—(1951*b*) *Ibid.*, **22**, 88.
Reingold, I. M., Ottoman, R. E., and Konwaler, B. E.—(1950) *Amer. J. clin. Path.*, **20**, 515.
Sellors, T. Holmes.—(1953) *Proc. Roy. Soc. Med.*, **46**, 861.
Simpson, S. L.—(1929) *Quart. J. Med.*, **22**, 413.
Willis, R. A.—(1952) "The Pathology of Tumours." London (Butterworth).